# Improving the Deaf community's access to prostate and testicular cancer information: a survey study

**DOI:** 10.1186/1471-2458-5-63

**Published:** 2005-06-06

**Authors:** Ann Folkins, Georgia Robins Sadler, Celine Ko, Patricia Branz, Shane Marsh, Michael Bovee

**Affiliations:** 1 University of California, San Diego, School of Medicine, 9500 Gilman Drive, La Jolla, California, 92093-0606, USA; 2 Rebecca and John Moores UCSD Cancer Center, University of California, San Diego, 9500 Gilman Drive, La Jolla, California, 92093-0658, USA; 3 San Diego State University/University of California, San Diego Joint Doctoral Program, 6363 Alvarado Court, Suite 103, San Diego, California, 92120-4913, USA; 4 Deaf Community Services of San Diego, Inc., 3930 4^th ^Avenue, Suite 300, San Diego, California, 92103, USA; 5 Bovee Productions, 2975 Palm Street, San Diego, California, 92104, USA

## Abstract

**Background:**

Members of the Deaf community face communication barriers to accessing health information. To resolve these inequalities, educational programs must be designed in the appropriate format and language to meet their needs.

**Methods:**

Deaf men (102) were surveyed before, immediately following, and two months after viewing a 52-minute prostate and testicular cancer video in American Sign Language (ASL) with open text captioning and voice overlay. To provide the Deaf community with information equivalent to that available to the hearing community, the video addressed two cancer topics in depth. While the inclusion of two cancer topics lengthened the video, it was anticipated to reduce redundancy and encourage men of diverse ages to learn in a supportive, culturally aligned environment while also covering more topics within the partnership's limited budget. Survey data were analyzed to evaluate the video's impact on viewers' pre- and post-intervention understanding of prostate and testicular cancers, as well as respondents' satisfaction with the video, exposure to and use of early detection services, and sources of cancer information.

**Results:**

From baseline to immediately post-intervention, participants' overall knowledge increased significantly, and this gain was maintained at the two-month follow-up. Men of diverse ages were successfully recruited, and this worked effectively as a support group. However, combining two complex cancer topics, in depth, in one video appeared to make it more difficult for participants to retain as many relevant details specific to each cancer. Participants related that there was so much information that they would need to watch the video more than once to understand each topic fully. When surveyed about their best sources of health information, participants ranked doctors first and showed a preference for active rather than passive methods of learning.

**Conclusion:**

After viewing this ASL video, participants showed significant increases in cancer understanding, and the effects remained significant at the two-month follow-up. However, to achieve maximum learning in a single training session, only one topic should be covered in future educational videos.

## Background

Interpersonal communication problems are present throughout the health care system. These problems take on greater significance when compounded by language and cultural barriers. The Deaf community, whose predominant language is American Sign Language (ASL), faces the same barriers to accessing health information and services as other communities whose members use English as a second language [1-22]. In a prior research study with the Deaf community, study participants reported that educational materials needed to be culturally and linguistically aligned if they are to optimally address the health disparities pervasive in the Deaf community [7]. The mode with which health information reaches the Deaf community is key to its value and impact. Given that this community relies heavily on the visual receipt of information, programs that are presented in ASL and enriched with open captioning and pictures are the best formats in which to distribute information [6,16,23-26].

This paper reports on the evaluation of a prostate and testicular cancer education video, filmed in ASL, to increase Deaf men's access to health information. Prostate cancer is the most common, life-threatening cancer in older American men (age 50 and older), and testicular cancer is a potentially curable disease that affects younger men (ages 15–40) [27]. Deaf men's empowerment and self-advocacy in the health care setting begins with greater access to health information.

## Methods

The 52-minute video used for this study, entitled *Prostate and Testicular Cancer: Know your Options*, was produced in 2002 through a partnership composed of Deaf Community Services of San Diego, Inc. (DCS), the Rebecca and John Moores UCSD Cancer Center, Bovee Productions, and Gallaudet University. The film was a New York Festivals Certificate of Distinction Finalist Winner in 2004. The educational video, filmed in ASL, shows two trainers providing answers to their audience's questions. Although the focus of this study was on the Deaf community, the addition of open text captioning and voice overlay without ambient music enabled the video to be accessed by both deaf and hard-of-hearing individuals, as well as their hearing loved ones. The video can be viewed at .

Participants in the authors' prior studies had recommended that the ASL videos should include the same amount and complexity of information as that which would be available to the hearing community. Further, they recommended that men, like women, needed access to quality information about their gender specific cancers. In the light of the demonstrated need for increased access to health information, the authors opted to create a video that addressed prostate cancer and testicular cancer. This offered a way of reducing redundancy, encouraging men of diverse ages to interact in a supportive, culturally aligned, learning environment, and also covered more topics on the partnership's limited budget. As a result, the final video was complex, particularly in light of the community's limited prior exposure to cancer information and the many new medical terms and concepts that needed to be introduced. As such, the film was intended for multiple viewings to achieve mastery all of the information. The study looked at the impact of only a single viewing.

Using a train-the-trainers model, the video was presented to small groups of Deaf men and women in community settings. Recruitment strategies were accomplished in collaboration with Deaf community service agencies throughout California: Deaf Community Services of San Diego, Inc.; Center on Deafness-Inland Empire; Deaf and Hard of Hearing Service Center, Inc.; Deaf Counseling, Advocacy and Referral Agency; NorCal Center on Deafness; Greater Los Angeles Agency on Deafness, Inc. These strategies included in-person and e-mailed word-of-mouth dissemination and IRB-approved flyers [28,29]. Study prospects were told that participants would be helping to evaluate a prostate and testicular cancer education program that had been specifically designed to improve the Deaf community's access to health information. As a thank you for their participation, a meal was provided to all members of the audience before the presentation and a $25 incentive was offered to those who completed all three survey documents. Participants were also given their own copy of the video and encouraged to view it again and share it with other members of the Deaf community.

After passing a knowledge competency exam, six native signers were trained how to use the educational video. Sixteen educational sessions were held with a total of 102 men and 59 women attending. Attendance per session ranged from five to 32 persons. Only men were invited to take part in the evaluation of the video, and of those 102 men who attended the sessions, all consented to participate. All participants were offered the option of having the consent process, the consent documents, and the surveys translated into ASL, a service required to varying degrees. The consented participants completed the baseline survey before viewing the video. The video had been filmed in a question and answer format to make it easy for the trainers to stop and start the video to stimulate audience discussion and assure comprehension of content. Study participants and other attendees were to watch the video under the direction of the local grassroots trainers with the oversight of the project coordinator, both native signers. At the conclusion of the video, participants completed post-education intervention surveys. Participants and other audience members (both male and female) then engaged in a focus group discussion lead by the facilitators, lasting approximately 30 minutes to 60 minutes. Two months later, follow-up meetings were set up via e-mail, TTY, or in-person for completion of the final survey, with 95 men (93% of original participants) completing the follow-up survey.

The paper and pen surveys collected socio-demographic data and used multiple-choice, true or false, and open-ended questions related to the presentation. All data collection instruments were approved by the University's Institutional Review Board (IRB).

### Statistical analysis

The surveys were analyzed using t-tests and McNemar chi-square tests to assess for differences between pre, post, and two-month responses. Comparisons made between the pre/post video surveys and the two-month follow-up surveys examined only the 95 men who responded at two months. Unanswered questions were omitted from the analysis.

### Sample description

The study participants all resided in Southern California and were between 18 and 86 years of age (mean age 44.35; SD 17.39). Participants completed an average of 13.68 (SD 3.32) years of school with a range from four years to 24 years. While 28.4% (29) of the group completed high school, 28.4% (29) completed some college, 20.6% (21) completed college, 14.7% (15) completed education beyond college, and 6.9% (7) did not answer the question. The group was composed of: 62.7% (64) Caucasians; 7.8% (8) African Americans; 5.9% (6) Asian/Pacific Islanders; 16.7% (17) Hispanics; 2.9% (3) Mixed; and 3.9% (4) Other. For health care expenses, 6.9% (7) paid for their health care out of pocket; the others had MediCal/Medicaid (27.5% (28)), Medicare (25.5% (26)), other health insurance (39.2% (40)), or other sources (1% (1)). Of the sample, 43.1% (44) reported having friends with prostate cancer, and 19.6% (20) reported having friends with testicular cancer. There were seven men (6.9%) who reported having had prostate cancer themselves, and there was one man (1%) who reported having had testicular cancer.

## Results

Table 1 shows the health care providers' educational interventions recalled by the participants. When asked about screening for prostate cancer, 26.1% (18/69) of the men under age 50 and 75.8% (25/33) of the men over age 50 reported that they had been examined by their doctor at some time for prostate cancer. When asked about screening for testicular cancer, 17.6% (6/34) of the men under age 35 and 33.9% (21/62) of the men over age 35 reported having been examined.

**Table 1:** Reported educational efforts of participants' health care providers

**Question**	**Responded Yes**
Has your doctor ever talked with you about prostate cancer?	27 (26.5%)
Has your doctor ever talked with you about testicular cancer?	13 (12.7%)
Has another health care provider ever talked with you about prostate cancer?	13 (12.7%)
Has another health care provider ever talked with you about testicular cancer?	6 (5.9%)
Have you been trained by a health care provider to do a testicular self-exam?	9 (8.8%)
Do you know how to do a testicular self-exam?	24 (23.5%)

When the men were asked whether they felt that there needed to be more programs on cancer and other health concerns specifically made for the Deaf community, 94.1% (96) responded affirmatively. Before and after the educational intervention, the participants were asked to rate their perception of the Deaf community's access to health information on a one to five scale with one being "very little" access and five being "a lot" of access. The results, shown in Table 2, reveal that most men rated health information access as "very little" or "little" before the video was shown, but perceived access to be higher after viewing the video, a statistically significant shift.

**Table 2:** Perception of access to health information on 1–5 scale (N = 102) (1-Very little, 2-Little, 3-Some, 4-Quite a bit, 5-A lot)

	Pre-test (mean) ± SD	Post-test (mean) ± SD
Prostate Cancer	2.06 ± 1.10	3.06* ± 1.53
Testicular Cancer	1.88 ± 1.04	3.01* ± 1.54

*T-test showed significant increase in mean score between pre and post-test surveys.

A total of 25 knowledge questions were asked on the surveys to assess whether there was any significant change in the participants' knowledge about prostate and testicular cancer from pre- to post-intervention and whether this increase was maintained at the two-month time point. From baseline to immediately post-intervention, participants' overall knowledge increased significantly (pre-video X_1 _= 15.2, post-video X_2 _= 18.4, t = -9.698, p < 0.05). X denotes the mean number of correct responses; all the participants' scores (number correct/25) were added, and the total was divided by the number of participants. From baseline to the two-month follow-up, participants maintained their overall statistically significant increase in knowledge (pre-video X_1 _= 15.3, two-month X_3 _= 17.1, t = -4.726, p < 0.05). These questions are clustered by format, and the results are shown in Tables 3 and 4.

**Table 3:** Percentage of correct responses to true or false questions

**Testicular true-false statements**	**Pre-video**	**Post-video**	**2 month post**
Testicular cancer usually occurs in men 15–40 years old. (True)	48 (47.5%)	94 (93.1%)*	80 (84.2%)^
Older men are more likely to get testicular cancer than younger men. (False)	34 (33.7%)	78 (77.2%)*	56 (58.9%)^
Testicular cancer can be cured. (True)	59 (59%)	68 (68%)	70 (73.7%)^
After treatment for testicular cancer, most men can still have children. (True)	49 (49.5%)	84 (84.8%)*	65 (69.9%)^
Your testicle needs to be removed if you have testicular cancer. (True)	73 (73%)	92 (92%)*	76 (80%)
When testicular cancer is suspected, a biopsy will be recommended. (False)	17 (16.8%)	43 (42.6%)*	29 (30.9%)
If testicular cancer is found in one testicle, the doctor will remove both testicles. (False)	70 (72.2%)	72 (72.4%)	75 (83.3%)
**Prostate true-false statements**			
Benign prostatic hyperplasia is a type of prostate cancer. (False)	41 (41%)	38 (38%)	34 (37%)
Older men are more likely to get prostate cancer than younger men. (True)	84 (82.4%)	89 (87.3%)	78 (82.1%)
"Watchful waiting" is an option for some cases of prostate cancer. (True)	50 (50%)	85 (85%)*	57 (60%)
Early detection of prostate cancer increases your treatment options. (True)	85 (84.2%)	92 (91.1%)	86 (90.5%)
When prostate cancer is suspected, a biopsy is recommended. (True)	80 (79.2%)	85 (84.2%)	75 (78.9%)
Men who are at high risk of getting prostate cancer should be offered screening every year beginning at age 45. (True)	79 (78.2%)	66 (65.3%)*	67 (70.5%)
Men who are at average risk of getting prostate cancer should be offered screening every year beginning at age 50. (True)	66 (65.3%)	94 (93.1%)*	74 (78.7%)^

*Significant increase in knowledge between pre- and post-test using McNemar chi -square test, alpha<0.05

^Significant increase in knowledge maintained between pre- and two-month test using McNemar chi-square test, alpha<0.05

**Table 4:** Ability to select correct multiple choice answers about prostate and testicular cancers

**Question**	**Pre-video**	**Post-video**	**2 month post**
**What is cancer?**			
A disease where your cells become mutated and divide uncontrollably.	73 (74.5%)	88 (89.8%)*	78 (83%)
**What is a Prostate Specific Antigen Test?**			
A test to measure the amount of PSA in a man's blood.	56 (56%)	70 (70%)*	62 (66.7%)
**What increases your risk of getting prostate cancer?**			
Getting older.	78 (78%)	76 (76%)	70 (74.5%)
Being White or African American.	25 (25%)	71 (71%)*	56 (59.6%)^
Family history of prostate cancer.	60 (60%)	79 (79%)*	68 (72.3%)
**What is a Digital Rectal Examination (DRE)?**			
An examination of a man's prostate through his rectum.	66 (67.3%)	79 (80.6%)*	66 (70.2%)
**Which of the following are possible side effects of treatment for prostate cancer?**			
Losing one's ability to have an erection.	58 (57.4%)	79 (78.2%)*	66 (70.2%)^
Losing one's ability to hold urine (pee).	75 (74.3%)	87 (86.1%)*	73 (77.7%)
**What increases your risk of getting testicular cancer?**			
Family history of testicular cancer.	63 (63%)	81 (81%)*	68 (73.1%)
Undescended testicle in childhood.	18 (18%)	47 (47%)*	35 (37.6%)^
**Which of the following is the treatment of testicular cancer?**			
Surgical removal of the testicle that contains the cancer.	72 (72%)	82 (82%)	71 (77.2%)

*Significant increase in knowledge between pre- and post-test using McNemar chi -square test, alpha<0.05

^Significant increase in knowledge maintained between pre- and two-month test using McNemar chi-square test, alpha<0.05

When the individual questions were analyzed, significant changes were also noted among the three time points. Of the seven true or false questions dealing with testicular cancer displayed in Table 3, five showed a statistically significant improvement in the frequency of correct responses immediately post-intervention. At two months, participants' responses showed that they maintained their increased knowledge from baseline for three of these five questions. Knowledge related to a sixth question (Testicular cancer can be cured) continued to increase at each time point and became significant at two months. As is evident from Table 3, some the of the most striking improvements in participants' understanding related to the age ranges during which men are at risk for testicular cancer and the consequences of treatment on fertility.

Table 3 also shows the results of seven true or false questions for prostate cancer. In contrast to baseline testicular cancer knowledge, baseline knowledge of prostate cancer was generally much higher. As a result, there was less room for improvement. Scores significantly increased from the pre-video to the post-video in only three of the individual questions and remained significantly higher for only one. After viewing the video, 85% (85) of the men correctly identified "watchful waiting" as an option for managing prostate cancer, representing a significant gain in knowledge. However, this post-test gain dropped to 60% (57) at two months, a value that was not significantly different than that of the pre-test survey. In addition, while most men seem to have learned that prostate screening is recommended for men of average risk beginning at 50 years old, they were not able to recall that men of higher risk should begin screening at age 45. The other three questions did not show a significant increase in knowledge, and it was clear that both before and after the video, most men were confused about the definition of Benign Prostatic Hyperplasia (BPH).

As shown in Table 4, nine of the 11 multiple-choice questions showed a statistically significant increase in knowledge immediately following the video presentation. For example, 47% of participants correctly acknowledged, after viewing the video, that having an undescended testicle is a risk factor for testicular cancer, an increase from 18% at baseline. The remaining two questions showed a high rate of correct responses before and after the video, indicating that the participants were already informed about these concepts. While there was generally still greater knowledge at two months than at baseline, the increase remained statistically significant for only three questions. Only for the question related to aging as a risk factor for prostate cancer was there a decrease in the number of correct responses from baseline to two months.

The participants were asked to rank order their best sources of health information from a list including the following: doctor, nurse, friends, family, newspaper, TV, magazine, health books/pamphlets, Internet, DCS, and special health education programs. The results shown in Table 5 reveal that while doctors remain the number one trusted source of information in this group, other sources like DCS, special health education programs, and the Internet are also important avenues of communication. Of note, written sources of information were not ranked as highly as person-to-person sources of information. Each box in Table 5 shows the percentage ranking of the top four responses for the first, second, third, and fourth response categories.

**Table 5:** Best sources of health information

**Rank**	**Pre-video**	**Post-video**	**2 month post**
First	**Doctor (47%)** DCS (15.7%) Internet (10.8%) Health education programs (9.8%)	**Doctor (48%)** DCS (16%) Internet (13.7%) Health education programs (9.8%)	**Doctor (42.2%)** DCS (18.6%) Internet (13.7%) Health education programs (7.8%)
Second	**Internet (18.6%)** DCS (15.7%) Health education programs (14.7%) Health books/pamphlets (7.8%)	**DCS (21%)** Internet (15%) Health books/pamphlets (12.7%) Doctor (12.7%)	**DCS (17.6%)** Doctor (15.7%) Health education programs (12.7%) Internet (9.8%)
Third	**DCS (17.6 %)** Health books/pamphlets (16.7%) Family (12.7%) Doctor (11.8%)	**Health education programs (19.6%)** DCS (15%) Health books/pamphlets (12.7%) Friends (12.7%)	**DCS (16.7%)** Health education programs (14.7%) Health books/pamphlets (11.8%) Internet (11.8%)
Fourth	**Health education programs (13.7%)** **Doctor (13.7%)** Friends and family (11.8%) Health books/ pamphlets (11.8%)	**Health education programs (15.7%)** **Family (15.7%)** DCS (14.7%) Health books/pamphlets (11.8%)	**Health education programs (20.6%)** DCS (11.8%) Doctor (11.8%) Health books/pamphlets (10.8%)

Participants also completed an open-ended question that asked them to list up to four sources of cancer information. Of the 102 participants, 76 listed at least one source, 48 listed at least two sources, 30 listed at least three sources, and 10 listed four sources. The results of these responses were coded according to whether English literacy was required and according to whether the learning was based on social interaction. For example, pamphlets, Internet, and books were coded as non-interactive, English language based methods of learning, while doctors, DCS, friends, and family were coded as interactive, socially based methods of learning. Analysis revealed an overwhelming preference for the latter. Seventy-one percent listed an interactive source of cancer information for response one, 69% for response two, 67% for response three, and 60% for response four. The results given above are taken from the baseline pre-video survey; the results from the same question in the post-video and the two-month surveys were not statistically different.

In the immediate post-intervention survey, 98 (96.1%) felt that the video provided useful information, but 88 (86.3%) felt that too much information was provided. Most of the men (85 (83.3%)) agreed that the video needed to be watched more than once to understand all the information, and 92 (90.2%) of the men planned on watching the video again. Seventy-six (74.5%) felt that most in the Deaf community would be interested in the video, and 95 (93.1%) were comfortable showing the video to their friends. At the two-month survey, 67 (65.7%) of the participants reported they had watched the video again, and 28 (27.5%) had already shared the video with someone else.

## Discussion

The goals of this study were to gain knowledge of participants' baseline screening behaviors and knowledge, evaluate the effectiveness of a prostate and testicular cancer video program in increasing knowledge about these cancers in the Deaf community, and gain feedback from the participants about their experience with the program and with other cancer information sources. An additional aim was to increase the Deaf community's awareness of available health resources.

This study demonstrates that the provision of culturally and linguistically aligned educational strategies can increase the community's access to health information. There was near universal agreement that the video provided useful information and that there needs to be more special health programs designed for the Deaf community. Most men viewed the video again, and many shared it with others. After viewing the video, which provided websites and TTY numbers to access additional information, and learning that the video could be accessed on the Internet, participants perceived that the Deaf community had increased access to health information. Consensus reached in the focus group discussions was that the video was a culturally aligned method of education about these two cancer topics. Thus, the participants' perception of an increase in access to information was the result of having information available in ASL and having knowledge of where to find additional resources.

Participants also demonstrated a significant increase in knowledge after viewing the video just once. Overall, there was an increase in the number of correct responses after the video in sixteen of the 25 knowledge questions. While this gain was not fully maintained at two months, it still remained above baseline at a statistically significant level. Since the men all received copies of the video and were aware of the video's presence on the Internet, they also knew they could access the information again to review or to deal with a health related crisis.

While including as much information in this video as possible was an admirable goal, given the dearth of information available in ASL, participants' feedback suggests that a single health topic focus would be better for future videos. Most of the participants felt that the video offered too much information to absorb in a single viewing. This is not surprising, given that the video attempted to explain highly complex scientific concepts like chemotherapy, metastasis, benign prostatic hypertrophy, and malignancy to a non-medical audience. While the video's focus on two cancers may have reduced participants' long-term retention of details, it allowed men of diverse ages to lend support and encouragement to each other during the training sessions and facilitated subject recruitment. Because the video was filmed as question and answer dyads, it is possible to view the two topics, prostate cancer and testicular cancer, separately. Another study is currently underway to explore the impact of just viewing the video dyads related to testicular cancer.

This feedback highlights the need for programs that continually reinforce health concepts in the Deaf community rather than just one-time seminars. This study also reinforces the importance of long-term follow-up in the assessment of educational interventions. Studies that simply survey participants before and after the intervention fail to recognize that long-term retention of information does not always correlate with short-term learning. Moreover, this study underscores the challenge health care providers face even when trying to explain complex medical issues through a medical interpreter. Videos such as this one should help to bridge the communication gaps.

While a strength of this study is its diverse sample, generalizations must still be drawn with caution since this group of men collectively had a relatively high level of education. It is unclear whether this video and educational format would be equally effective for deaf persons who have a low ASL literacy or more limited education. Additional evaluation of this educational format is therefore warranted with those audiences. Further, as with all studies, these men were self selected by their interest in learning about cancer, so their motivation to gain knowledge was probably higher than average. They may not be representative of all patients who will present for medical care.

The most popular sources of information among the surveyed individuals included physicians, Internet, DCS, and health education programs. The available literature reports communication problems and mistrust between deaf and hard-of-hearing persons and their health care providers [13,21,30,31]. In contrast, the findings in this study show that most of the men rely on their doctors for health information. They also report a high frequency of prostate exams and a relatively high knowledge of prostate cancer information at baseline. Thus while there may be a general distrust between the Deaf community and health care providers, these participants and their health care professionals are clearly succeeding at overcoming at least a proportion of the barriers to information and health care. Alternately, while the participants are accessing this information and care, they may still be reporting the difficulties they experience in achieving these outcomes.

This educational intervention was created specifically to help health care providers meet the information needs of the Deaf community. Since the video can be viewed on the Internet at the Rebecca and John Moores UCSD Cancer Center website  and through the National Association of the Deaf's Caption Media Resource library, health care providers can encourage their patients to view this and other videos provided in ASL or with captioning. While such educational resources will offer a valuable tool to providers and their deaf patients, the focus group discussions showed that the provision of high level certified medical interpreters would be the single most important improvement health care providers could offer to their deaf patients to reduce the difficulties they experience when seeking medical care. With the growing number of certified medical interpreters and technology advances that have made video relay services available, it should be increasingly easier for physicians and their patients to access high level interpreting services more efficiently.

When the participants' responses regarding sources of cancer information were analyzed according to whether English literacy was required and according to whether the learning was based on social interaction, participants overwhelmingly preferred interactive forms of learning. This further reinforces the importance of providing information in the correct format to properly meet the needs of Deaf community. Ideally, an ASL video should be accompanied by person-to-person interaction between the viewer and another individual familiar with the health topic in order to facilitate better retention and understanding of the material. Television was rarely mentioned and radio was never mentioned as a source of information; this highlights the fact that these popular sources of information for the hearing public are not adequate means to address the needs of the Deaf community.

An unforeseen benefit of this program for the Deaf community was that it exposed many individuals to the process of research studies and allowed them to become familiar with the consenting process, data collection procedures, and protocol adherence. Considerable time was devoted to explaining the reasons why one does a pre- and post-survey and how the results of these surveys could be used to design even better programs for the Deaf community. Study coordinators remarked on the audience's enthusiasm about understanding how the video was made and how the research study worked. They were uniformly appreciative of the intervention and willing to share the experience with loved ones and friends outside of the sessions. In the future, this positive exposure may encourage members of the Deaf community to consider invitations to participate in clinical trials and other research studies.

## Conclusion

ASL videos provide an effective tool for bringing cancer information to the Deaf community. Deaf men in the study showed significant increases in their short and long-term understanding of prostate and testicular cancers after viewing the film, *Prostate and Testicular Cancer: Know Your Options*. At the two-month follow-up survey, this increase in knowledge was still significant, although slightly diminished. This relative lack of sustainability is due in part to the complexity of the material, but it also highlights that multiple viewings are needed to gain the full benefit of the educational intervention. The Deaf community, like other communities, will benefit from being exposed to multiple repetitions of the same message. Future videos should be focused on one cancer and shorter in duration. The men in the study confirmed the need for more health education materials aimed at the Deaf community and rated doctors as their number one source of health information. Ideally, increasing distribution of health education materials to physician's offices would facilitate better access to and trust in educational interventions.

## Competing interests

The author declares that he has no competing interests.

## Authors' contributions

AF wrote the script and designed the graphics for the video, participated in the data analysis, and drafted the manuscript. GRS conceived the study, oversaw the day-to-day operation of the project, and drafted the manuscript. CK performed the statistical analysis and assisted with review of the manuscript. PB and SM were co-leaders in the study's training sessions, conducted the informed consenting process, collected and managed the database, and assisted with the cultural interpretation of the results. MB produced the video, and assisted with review of the manuscript. All authors have read and approved the final manuscript.

## Pre-publication history

The pre-publication history for this paper can be accessed here:


